# Recent trends in robot learning and evolution for swarm robotics

**DOI:** 10.3389/frobt.2023.1134841

**Published:** 2023-04-24

**Authors:** Jonas Kuckling

**Affiliations:** IRIDIA, Université Libre de Bruxelles, Brussels, Belgium

**Keywords:** swarm robotics, robot evolution, robot learning, automatic design, neuro-evolution, automatic modular design, embodied evolution, imitation learning

## Abstract

Swarm robotics is a promising approach to control large groups of robots. However, designing the individual behavior of the robots so that a desired collective behavior emerges is still a major challenge. In recent years, many advances in the automatic design of control software for robot swarms have been made, thus making automatic design a promising tool to address this challenge. In this article, I highlight and discuss recent advances and trends in offline robot evolution, embodied evolution, and offline robot learning for swarm robotics. For each approach, I describe recent design methods of interest, and commonly encountered challenges. In addition to the review, I provide a perspective on recent trends and discuss how they might influence future research to help address the remaining challenges of designing robot swarms.

## 1 Introduction

Robot swarms are decentralized systems of relatively simple robots that only rely on local information to operate (Beni, 2005; Şahin, 2005; Brambilla et al., 2013; Dorigo et al., 2014; Hamann, 2018). Like animal swarms in nature, a robot swarm is a group of robots that are efficient at performing tasks due to their cooperation. Robot swarms are multi-robot systems that exhibit some particular characteristics. They are decentralized and highly redundant. The high redundancy requires that there is no role in the swarm that can only be executed by a single robot^1^. Furthermore, in a robot swarm, there exists no single central point of control (neither internal nor external to the swarm), as a centralized point of control would be a single point of failure. Therefore, complex collective behaviors, such as task allocation, cannot be planned and orchestrated by an operator. Instead, the swarm is required to be self-organizing: the collective behavior of the swarm must emerge from the interactions between the individual robots. Additionally, the robots in the swarm are relatively simple (both in terms of hardware and software) with respect to the task they perform and have only local sensing and communication capabilities.

These inherent characteristics of robot swarms promote the development and implementation of robotic systems that exhibit desirable properties (Şahin, 2005; Dorigo et al., 2014; Hamann, 2018). The self-organized nature of robot swarms promotes the design of flexible systems: the swarm can adapt to different and potentially changing environments. Additionally, the redundancy of the swarm facilitates the creation of systems that are fault tolerant. The failure of any individual robot (or sometimes significant portions of the swarm) will not prevent the swarm from achieving its task. Lastly, as robots only interact with their immediate neighboring peers, swarms are scalable systems. That is, the addition or removal of robots from the swarm does not significantly affect the performance of the swarm. Thanks to these properties, swarm robotics is considered a prominent approach to control large groups of autonomous robots (Rubenstein et al., 2014; Werfel et al., 2014; Mathews et al., 2017; Garattoni and Birattari, 2018; Slavkov et al., 2018; Yu et al., 2018; Li et al., 2019; Xie et al., 2019; Dorigo et al., 2020) and has been recently highlighted as one of the grand challenges of robotics research for the upcoming years (Yang et al., 2018). The use of robot swarms has been proposed for coordinating groups of robots in missions in dynamic or unknown environments, such as for space exploration, search and retrieval in disaster situations, or agricultural applications (Carrillo-Zapata et al., 2020; Dorigo et al., 2020). While no real-world application of swarm robotics exists yet, they are projected to be developed within the next ten to 15 years (Dorigo et al., 2020).

Although the realization of robot swarms offers several advantages, their decentralized and self-organized nature makes them challenging to design. The requirements for the desired behavior of the swarm are usually expressed at the collective level, but it is not possible to program the swarm directly. Instead, the individual robots need to be programmed in such a way that the desired collective behavior arises. The problem is that each robot can only act based on the local information that it can perceive. When programming the robots, the designer needs to predict how the local behaviors and local interactions between robots will contribute to the emergence of the desired collective behavior.

Swarm robotics originated from the application of bio-inspired swarm intelligence principles to robotics (Beckers et al., 1994; Beni, 2005). Since then, swarm robotics has moved towards a more matured engineering discipline—often referred to as swarm engineering (Winfield et al., 2005; Brambilla et al., 2013). Swarm engineering concerns the creation of arbitrary (not necessarily bio-inspired) collective behaviors for a robot swarm. The most common approach to the design of robot swarms is manual design: a human designer manually implements the control software for the robots. The designer can refine the control software through a trial-and-error process until they find the result satisfactory. While this design process often yields reasonably good results, it can be error-prone, costly, time-consuming and the quality of the control software strongly depends on the expertise of the designer. Furthermore, there is no guarantee that the performance will reach a satisfactory level within any reasonably available time budget. Several principled methods and design patterns for designing collective behaviors have been developed to overcome the limitations of pure trial-and-error design (Halloy et al., 2007; Soysal and Şahin, 2007; Hamann and Wörn, 2008; Yamins and Nagpal, 2008; Berman et al., 2009; Kazadi, 2009; Prorok et al., 2009; Berman et al., 2011; Beal et al., 2012; Brambilla et al., 2014; Reina et al., 2015; Lopes et al., 2016; Pinciroli and Beltrame, 2016; Hamann, 2018). Yet, these methods are restricted to specific assumptions and no generally applicable methodology has been proposed yet (Brambilla et al., 2013; Francesca and Birattari, 2016; Schranz et al., 2021).

An alternative to principled methods are (semi-)automatic design methods, which are built upon techniques such as robot evolution or robot learning. In semi-automatic design, a human designer remains in the loop during the development and optimization of the control software. That is, the human designer can observe and intervene in the design process, if necessary. For example, the human designer could observe the result found by the design process, change some parameters used in the algorithms to produce the control software and restart them with the new parameter values. The semi-automatic design terminates when the human designer is satisfied with the generated control software. In a research setting, semi-automatic design allows to assess the underlying feasibility of these design methods. However, in practice, semi-automatic design exhibits similar drawbacks as manual design. Namely, the quality of the generated control software depends on the human designer and their ability to steer the design process. In fully automatic design (Birattari et al., 2019), no human intervention beyond the mission specification is possible (Birattari et al., 2020). That is, the design process runs completely automatic until it terminates with the generation of an appropriate instance of control software. (Semi-)Automatic design methods can be further categorized into online and offline methods (Bredeche et al., 2018; Birattari et al., 2020). In online design, the design process is executed while the swarm performs its mission in the target environment, whereas in offline design, the design process is executed before the swarm is deployed to perform its mission.

In this work, I discuss recent advances in robot evolution and robot learning in the context of swarm robotics (see Tables 1–7 for an index of the considered design methods). The work is organized as follows. In Section 2, I present offline design methods that rely on evolutionary algorithms or related techniques. In Section 3, I present online design methods. In Section 4, I present offline design methods based on robot learning. In Section 5, I provide a perspective on important open questions on the application of robot learning and evolution in swarm robotics.

**TABLE 1:** Overview of selected neuro-evolutionary research in swarm robotics.

Publication	Swarm composition	Mission	Network topology	Algorithm	Sim.	Real.
Trianni and Nolfi (2011)	3 s-bot robots	Synchronization	Single layer perceptron	Evolutionary algorithm	•	
Gauci et al. (2014a)	10 e-puck robots	Aggregation	Fully-recurrent network	Classical Evolutionary Programming	•	•
Duarte et al. (2016)	5–10 aquatic drones	Homing,	Feedforward network	NEAT	•	•
		dispersion,				
		clustering,				
		area monitoring				
Gomes et al. (2019)	1 aerial, 1 ground robot	Foraging	Feedforward network	NEAT	•	
Hasselmann et al. (2021)	20 e-puck robots	Aggregation,	Single layer perceptron,	CMA-ES, xNES, NEAT,	•	•
		homing	multi-layer perceptron	evolutionary algorithm		
		foraging				
		sheltering				
		gate passing				
van Diggelen et al. (2022)	14 ground robots	Gradient following	Fully connected reservoir	Differential evolution	•	
			network			

**TABLE 2:** Overview of selected automatic modular design research in swarm robotics.

Publication	Swarm composition	Mission	Architecture	Modules	Sim.	Real.
Hecker et al. (2012)	1–3 ground robots	Foraging	Finite-state machine	Manually implemented	•	•
Duarte et al. (2014)	50 aquatic drones	Patrolling	Finite-state machine	Evolved continuous-time	•	
				recurrent network		
Francesca et al. (2014)	20 e-puck robots	Aggregation,	Finite-state machine	Manually implemented	•	•
		foraging				
Ferrante et al. (2015)	4 foot-bot robots	Foraging	Rule set	Manually implemented	•	
Jones et al. (2018)	25 kilobot robots	Foraging	Behavior tree	Manually implemented	•	•
Neupane and Goodrich (2019)		Foraging,	Behavior tree	Manually implemented	•	
		co-op. transport,				
		nest maintenance				
Ligot et al. (2020a)	20 e-puck robots	Aggregation,	Finite-state machine	Evolved feedforward	•	•
		foraging		networks		

**TABLE 3:** Overview of selected novelty search research in swarm robotics.

Publication	Swarm composition	Mission	Sim.	Real.
Gomes et al. (2013)	5–7 e-puck robots	Aggregation, resource sharing	•	
Gomes et al. (2017)	3–8 ground robots	Predator-prey, herding,	•	
		cooperative foraging		
Gomes and Christensen (2018)	5–10 ground robots	Aggregation, clustering, coverage,	•	
		border coverage, dispersion,		
		phototaxis, flocking		
Hasselmann et al. (2023)	20 e-puck robots	Foraging, aggregation, sheltering	•	•

**TABLE 4:** Overview of selected other evolution-based research in swarm robotics.

Publication	Swarm composition	Mission	Approach	Sim.	Real.
Hamann (2014)	20 particles	Collective motion	Minimizing surprise	•	
Gauci et al. (2014b)	5–50 e-puck robots	Clustering	Computation-free control	•	•
Trianni and López-Ibáñez (2015)	6–10 foot-bot robots	Flocking,	Multi-objective optimization	•	
		collaboration			
Kaiser and Hamann (2019)	100 grid-world agents	Collective motion	Minimizing surprise	•	

**TABLE 5:** Overview of selected embodied evolution research in swarm robotics.

Publication	Swarm composition	Mission	Algorithm	Controller update	Sim.	Real.
Bianco and Nolfi (2004)	64 s-bot robots	Self-assembly	Not identified	Encounter, time-out	•	
Prieto et al. (2010)	8 e-puck robots	Cleaning	r-ASiCo	Energy		•
Bredeche et al. (2012)	9–100 e-puck robots	Foraging	mEDEA	Energy, time-out	•	•
Silva et al. (2015)	5 e-puck robots	Aggregation,	odNEAT	Energy	•	
		phototaxis,				
		collective motion				
Jones et al. (2019)	9 e-puck robots	Collective transport	Parallel island model	Time-out		•
			distributed evolution			
Cambier et al. (2021)	25–200 kilobot robots	Aggregation	Cultural evolution	Encounter	•	•

**TABLE 6:** Overview of selected multi-agent reinforcement learning research in swarm robotics.

Publication	Swarm composition	Mission	Algorithm	State-space	Sim.	Real.
Matarić (1997)	4 IS Robotics R2 robots	Foraging	Q-learning	Individual		•
Hüttenrauch et al. (2019)	2–10 ground robots	Rendez-vous,	Trust Region Policy Optimization	Individual	•	
		predator-prey				
Bloom et al. (2022)	4–8 foot-bot robots	Collective transport	ADAM	Individual	•	

**TABLE 7:** Overview of selected imitation learning research in swarm robotics.

Publication	Swarm composition	Mission	Algorithm	Demonstration	Sim.	Real.
Li et al. (2016)	5–11 e-puck robots	Aggregation,	Turing learning	Motion trajectories	•	•
		object clustering				
Šošić et al. (2017)	200 particles	Synchronization	Inverse reinforcement learning	Motion trajectories	•	
Alharthi et al. (2022)	20 ground robots	Not identified	Behavior cloning	Video recordings	•	
Gharbi et al. (2023)	20 e-puck robots	Aggregation,	Apprenticeship learning	Robot positions	•	•
		dispersion,				
		sheltering				

## 2 Robot evolution

The application of evolutionary robotics principles (Husbands and Harvey, 1992; Nolfi and Floreano, 2000) to swarm robotics is called *evolutionary swarm robotics* (Trianni, 2008; Nolfi, 2021). In evolutionary swarm robotics, the control software of the robots is generated through an artificial evolutionary process. Unless otherwise specified, the same generated control software is uploaded to each robot to be executed individually. The evolutionary process optimizes instances of control software with respect to a mission-specific *objective function*, often also called fitness function. The objective function is used to assess the quality of instances of control software, and in a way, provides selection pressure to direct the optimization process. Poorly performing instances are discarded and the well performing ones are *selected* to generate new instances through *recombination* and *mutation*. The methods presented in this section are automatic offline design methods. That is, methods in which the design process is executed in a centralized manner using simulations and before the robots are deployed. For design methods that run the evolutionary process directly on the robots, see Section 3.

In the context of swarm robotics, robot evolution is the most studied automatic design approach. Indeed, evolutionary swarm robotics has been used to create control software for robot swarms in a wide variety of mission such as foraging, collective transport, or pattern formation (Brambilla et al., 2013; Schranz et al., 2020). Traditionally, evolutionary swarm robotics has relied on *neuro-evolution*—the control software in the form of an artificial neural network is optimized using a centralized evolutionary algorithm (see Section 2.1). Other related approaches include *automatic modular design* (see Section 2.2) and *novelty-search-based design* (see Section 2.3). In automatic modular design, the control software is composed of modules that are assembled into more complex control architectures, such as finite-state machines or behavior trees. In novelty-search-based design methods, the selection pressure does not arise from the mission-specific objective function, but rather from a metric of behavioral novelty. While evolutionary swarm robotics design methods have demonstrated promising results in the past, they still face some important challenges that remain unsolved: notably, the generation of control software that is robust to the reality gap and the engineering of appropriate objective functions that can produce a desired collective behavior.

Trianni et al. (2014) and Francesca and Birattari (2016) provide overviews of robot evolution in the context of swarm robotics. For reviews of evolutionary robotics in the single-robot case, see Doncieux et al. (2011), Bongard (2013), Trianni (2014), Doncieux et al. (2015), and Silva et al. (2016).

### 2.1 Neuro-evolution

*Neuro-evolution*: Robots are controlled by an artificial neural network that maps sensor inputs to actuator outputs. The weights of the neural network, and possibly its topology, are optimized using an evolutionary algorithm with regard to a mission-specific objective function. The design process results in a single well-performing instance of control software.

Neuro-evolution is one of the earliest automatic design methods in swarm robotics (Dorigo et al., 2003; Quinn et al., 2003; Trianni et al., 2003). In this approach, neural networks are used as *black-box* controllers, and the search performed by the evolutionary algorithm does not require domain-specific heuristic information. For this reason, neuro-evolutionary design methods are expected to allow the design of control software with no domain knowledge. For a review of early neuro-evolutionary design methods, see Brambilla et al. (2013).

More recently, several authors have focused on systematically using neuro-evolution to design control software for various robotic platforms—mainly targeting those that could be possibly used in real-world deployments. For example, Trianni and Nolfi evolved a perceptron network to synchronize the movement of a swarm of s-bot robots (Trianni and Nolfi, 2011). Duarte et al. (2016) used NEAT (Stanley and Miikkulainen, 2002) to design control software a swarm of aquatic robots performing tasks such as homing or dispersion (Duarte et al., 2016). Gomes et al. (2019) generated control software for robot teams composed of aerial and ground robots in a foraging task. Hasselmann et al. (2021) compared NEAT, xNES (Glasmachers et al., 2010) and CMA-ES (Hansen and Ostermeier, 2001) to generate control software for a swarm of e-puck (Mondada et al., 2009) robots in five different missions such as aggregation, homing, shelter, foraging, and gate passing (Hasselmann et al., 2021). In a different research direction, researchers have investigated the minimal requirements to evolve specific collective behaviors. For example, Gauci et al. (2014a) evolved a recurrent neural network to perform aggregation. In their study, the authors tested their control software on robots with minimal capabilities: each robot had a single binary sensor that controlled the speed of its two wheels. van Diggelen et al. (2022) evolved a gradient following behavior. Notably, the robots could perceive only the local value of the gradient, not its direction, and they could not communicate with other robots.

Neuro-evolutionary approaches have shown many promising results. Yet, two main challenges remain in the field: fitness engineering and the reality gap. The first challenge is fitness engineering, or how to produce appropriate objective functions to drive the evolutionary process. It is well understood that incorrectly defined objective functions pose two challenges to the evolutionary process: *bootstrapping* and *deception* (Silva et al., 2016). The issue of bootstrapping arises when the objective function fails to apply meaningful selection pressure in low-performance regions of the search space. As a result, the design process explores the low-performance regions in an undirected manner and is unable to converge towards higher performance regions of the search space. The issue of deception describes the case in which the objective function contains easily reachable local optima. In this case, the design process can easily converge towards the local optima and will result in the generation of a suboptimal collective behavior. These two issues can usually be overcome by introducing *a priori* knowledge into the objective function (fitness engineering) (Trianni and Nolfi, 2011; Divband Soorati and Hamann, 2015; Silva et al., 2016). However, the necessity of *a priori* knowledge conditions the effectiveness of a neuro-evolutionary design method; as it will largely depend on the expertise of the designer of the objective function. The second challenge of neuro-evolution is the reality gap. The reality gap are the inescapable differences between the design and deployment environment, and often manifests in a performance drop when designing control software in simulation and assessing it on real robots. Yet, not all design methods are affected similarly by the reality gap, and it is therefore imperative to assess all automatic design methods not only in simulation but on real robots (Birattari et al., 2019). In the context of neuro-evolution, Hasselmann et al. investigated the effects of the reality gap on different neuro-evolutionary design methods (Hasselmann et al., 2021). They showed that, without further mitigation strategies or mission-specific adaptations, sophisticated neuro-evolutionary design methods perform similarly poor in reality as a simple perceptron network.

### 2.2 Automatic modular design

*Automatic modular design*: Robots are controlled by an instance of control software assembled from modules. Typical architectures of the control software include finite-state machines and behavior trees. An optimization algorithm possibly assembles the modules within the architecture and further fine-tunes the parameters of the modules, according to a mission-specific objective function. The design process results in a single well-performing instance of control software.

Neuro-evolution enables, in practice, the design of control software without prior domain knowledge. Yet, in cases that domain knowledge is available, it might be incorporated into the design method to achieve better results. Instead of relying on artificial neural networks, automatic modular design methods generate control software that is composed of software modules that are assembled into a more complex control architecture—e.g., finite-state machines or behavior trees (Colledanchise and Ögren, 2018). Through the choice and implementation of these modules, domain knowledge can be incorporated into the design process.

Duarte et al. (2014) manually decomposed a complex object removal task into simpler subtasks. They evolved continuous-time recurrent neural networks that were then assembled, in a modular way, into a hierarchical controller (according to the manual decomposition). Ferrante et al. (2015) used grammatical evolution to design control software for a foraging scenario with task allocation. They designed behavioral rules from basic behavioral and conditional modules. Hecker et al. (2012) used a genetic algorithm to optimize a finite-state machine that controls the behavior of robots in a foraging swarm. The authors pre-programmed an initial finite-state machine, which was inspired by the foraging behavior observed in ants. They used the genetic algorithm to optimize parameters of the finite-state machine that were not chosen at design time. Francesca et al. (2014) proposed AutoMoDe-Vanilla, an automatic modular design method that assembles finite-state machines out of a set of twelve handcrafted modules. Several flavors (i.e., implementations) of AutoMoDe have been proposed to study different elements of the design process, such as different module sets (Ligot et al., 2020a; Garzón Ramos and Birattari, 2020; Hasselmann and Birattari, 2020; Spaey et al., 2020; Mendiburu et al., 2022), hardware-software co-design (Salman et al., 2019), or optimization algorithms (Kuckling et al., 2020a; Kuckling et al., 2020b; Cambier and Ferrante, 2022). Besides finite-state machines, behavior trees have recently gained attention in the literature on automatic modular design. They offer several advantages over finite-state machines, like enhanced modularity and better human readability (Colledanchise and Ögren, 2018). Jones et al. (2018) evolved behavior trees for a foraging swarm of kilobot (Rubenstein et al., 2014) robots. Kuckling et al. investigated the use of behavior trees within the AutoMoDe framework (Kuckling et al., 2018; Kuckling et al., 2020a; Ligot et al., 2020b; Kuckling et al., 2022). Neupane and Goodrich used grammatical evolution to design software for a swarm of 100 robots performing a foraging task (Neupane and Goodrich, 2019).

Automatic modular design methods are an emerging field of research with promising prospects. Preliminary results indicate that they are a viable alternative to neuro-evolutionary design methods, with comparable performance and better transferability between simulation and real robots. However, this advantage comes at the cost of devoting effort to specify the modules. An artificial neural network can map all possible sensory inputs to all possible actuator outputs. As a result, neuro-evolutionary design methods can be used to design control software to perform any mission that is within the capabilities of the robots. In the case of automatic modular design, a human designer must manually implement the modules. The choice of modules implicitly restricts the space of possible missions that can be addressed by an automatic modular design method (Garzón Ramos and Birattari, 2020). If the set of modules is too limited, the design method would only produce satisfactory results for the mission it was conceived for, and the design space might not contain well-performing instances of control software for other missions. In other words, the design method will underperform in most cases. In this situation, the underperforming method can be accepted as it is or it will become necessary to develop a new design method—which ultimately turns into a manual design method rather than an automatic one. An important question to be addressed is, therefore, how to develop general automatic modular design methods that still remain robust to the reality gap.

### 2.3 Novelty search and quality diversity algorithms

*Novelty search*: Robots are controlled by an instance of control software in an arbitrary form, although commonly an artificial neural network is used. Instead of optimizing a mission-specific objective function, novelty search algorithms are selecting for control software that exhibits behavioral novelty with regard to previously encountered behaviors. The design process results in a set of behaviorally diverse instances of control software.

*Quality diversity algorithms*: Robots are controlled by an instance of control software in an arbitrary form. The design process considers two criteria: the quality with respect to a mission-specific objective function and the behavioral novelty with respect to previously encountered instances of control software. The design process returns either a single well-performing instance of control software or a set of diverse and relatively well-performing instances of control software.

Some recent studies focus on the application of novelty search in swarm robotics. Instead of optimizing a mission-specific performance measure, novelty search generates a set of behaviorally diverse instances of control software (Lehman and Stanley, 2011). This approach promises to avoid the issue of deception in objective function engineering. The design method avoids premature convergence by optimizing behavioral diversity instead of the mission-specific objective function.

Gomes et al. (2013) used novelty search to generate aggregation and resource sharing behaviors in a swarm. Additionally, the authors combined the novelty metric with a performance metric to overcome limitations where novelty search could not escape large, low-performance regions of the search space. In a follow-up work, Gomes et al. (2017) applied novelty search to co-evolutionary problems. Gomes and Christensen also investigated how to generate task-agnostic behavior repertoires using novelty search (Gomes and Christensen, 2018). Hasselmann et al. (2023) proposed AutoMoDe-Nata, an automatic modular design method that uses novelty search to create basic behavioral modules, which then are combined into probabilistic finite-state machines.

In the papers mentioned above, novelty search-based methods have shown to generate simple swarm robotics behaviors. Yet, for more complex collective behaviors, novelty search has not produced control software that performs as well as control software generated with a mission-specific objective function (Gomes and Christensen, 2018; Hasselmann et al., 2023). The outcome of novelty search methods is not a single instance of control software but a set of them—each of which is a behavior with sufficiently different traits. Therefore, the design method must also include a strategy to select the most appropriate behavior of the set (either manually or automatically). Quality diversity algorithms (Pugh et al., 2016) combine the benefits of novelty search with the directed search of evolutionary robotics. Beyond the difficulties of generating complex collective behaviors, novelty search further faces the challenge of defining characteristic traits that describe the collective behavior of the robots. So far, the selection of behavioral characteristics has been done *ad hoc* (Gomes et al., 2013; Gomes and Christensen, 2018; Hasselmann et al., 2023). However, this *ad hoc* selection requires expertise and it can result in potential drawbacks to the design process when done incorrectly. Vast behavior spaces defined by too general behavior characteristics will contain dimensions unrelated to the mission at hand, and they will cause the novelty search to perform poorly (Gomes et al., 2014). Furthermore, it remains open whether the behavioral characteristics of a collective behavior should be defined on a collective level, on an individual level, or with a combination of the two.

### 2.4 Other approaches

As discussed previously, a major issue in evolutionary swarm robotics is the definition of an appropriate objective function. Other researchers have addressed this issue besides those that focus on novelty search—although they are less prominent in the literature.

*Multi-objective optimization*: Robots are controlled by an instance of control software in an arbitrary form. Instead of optimizing a single mission-specific objective function, several objectives are considered at the same time. The design process results in a set of non-dominated instances of control software.

Trianni and López-Ibáñez (2015) investigated the use of an evolutionary multi-objective optimization algorithm in a strictly collaborative mission. Next to the (singular) objective of the mission, the authors specified a secondary auxiliary objective to overcome the convergence to certain sub-optimal behaviors—although the auxiliary conflicted with the main objective. They showed that multi-objective optimization indeed avoided premature convergence, and that properly chosen auxiliary objectives have the potential to overcome the bootstrap problem.

*Minimizing surprise*: Robots are controlled by two artificial neural networks. The first neural network is an action network that maps sensor inputs to actuator outputs; the other is a predictor network that maps sensor inputs to the predicted sensor inputs of the next control step. An evolutionary algorithm is used to optimize both neural networks together, minimizing only the prediction error of the predictor network. The design process results in a single instance of control software that minimizes the prediction error of the predictor network.

Kaiser and Hamann investigated an approach named “minimizing surprise.” Inspired by the *free energy principle* (Friston, 2010), Hamann used offline evolution to generate control software in the form of two neural networks, a prediction network and an action network (Hamann, 2014). The action network controlled the robot, whereas the prediction network predicted the next sensor state. The design process aimed to minimize the prediction error. Results showed that, despite not selecting for swarm behaviors, basic self-organizing collective behaviors emerged during the design process. Kaiser and Hamann extended their work and proposed a system to systematically engineer self-organizing assembly behaviors using the “minimizing surprise” approach (Kaiser and Hamann, 2019).

*Computation-free control*: Robots are controlled by a look-up table that contains the actuator output for every possible sensor input. The look-up table is optimized with respect to a mission-specific objective function. Typically, CMA-ES is used as optimization algorithm, but other algorithms are possible (e.g., exhaustive search). The design process results in a single well-performing instance of control software.

Gauci et al. (2014b) studied the emergence of collective behaviors for robots with minimal capabilities. In their study, the robots only had a single line-of-sight sensor and could set their velocity based on the discrete readings of this sensor. The authors used CMA-ES to optimize the mappings of the sensor to velocities in missions such as clustering, shepherding (Özdemir et al., 2017; Dosieah et al., 2022), decision making (Özdemir et al., 2018), and coverage (Özdemir et al., 2019).

The approaches described in this section are promising alternatives for the design of control software for robot swarms, but they require further research before they will become robust engineering techniques. Multi-objective design methods might overcome issues of deception and bootstrapping, similar to novelty search. However, they do not require the definition of behavioral characteristics. Instead, a secondary mission-specific objective is defined. This also poses a major challenge, as the definition of secondary objectives requires knowledge of any undesired behaviors. Minimizing surprise has shown to generate spatially organized behaviors in which the sensors states are stable. By defining partial expected sensor readings, generated instances of control software can be biased towards desired behaviors, yet other less desired behaviors were still generated. More research will be necessary to develop techniques to reliably generate desired behaviors (or classes of desired behaviors) *via* minimizing surprise. Similarly, computation-free control has been successful in some relatively simple missions, but further research will be necessary to show its viability in more complex missions.

## 3 Embodied evolution and social learning

*Embodied evolution*: Robots are controlled by instance of control software in an arbitrary form, though, typically, an artificial neural network is chosen. While performing the mission, the robots also run a decentralized evolutionary algorithm and periodically select a new instance of control software to execute. Repeatedly during the execution, the robots may exchange information about the performance of their executed instances of control software. The design process results in a single well-performing instance of control software.

Online design processes in which each robot in the swarm executes part of a distributed evolutionary algorithm are called embodied evolution. For example, in the design process, every robot in the swarm is initialized with and maintains its own set of genomes. From this genome set, each robot selects one genome and executes the control software encoded in it. Periodically, all robots exchange genomes among each other (which may be subjected to mutation or crossover operations) and they select a new genome to execute. Embodied evolution provides important advantages with respect to offline approaches (Watson et al., 2002). As evolution takes place directly in the mission environment, transferability is no concern. Besides, the distributed nature of the design process allows exploring different solutions in parallel. The parallelization of the design process allows to speed up the production of control software if compared with centralized online evolutionary methods.

Recent works (Heinerman et al., 2015; Silva et al., 2017; Bredeche and Fontbonne, 2021) have framed the concepts behind embodied evolution as a form of robot learning. The authors argue that embodied evolution is conceptually closer to robot learning (see Section 4), as the robots are updating their control software while performing the mission. Yet, the most common technique to implement embodied evolution remains the application of evolutionary algorithms.

Only a few studies have been conducted using embodied evolution in swarm robotics. For example, Bianco and Nolfi (2004) investigated an embodied evolutionary approach in which robots share their genomes when physically connecting to other robots. Prieto et al. (2010) used embodied evolution to program a swarm of e-puck robots in a cleaning task. Bredeche et al. (2012) investigated the adaptivity of open-ended evolution to changes in the environment. Silva et al. (2015) developed an online, distributed version of NEAT (Stanley and Miikkulainen, 2002) and used it to evolve control software in three missions. Jones et al. (2019) evolved behavior trees for a collective pushing task. Cambier et al. (2021) used an evolutionary language model to tune the parameters of a probabilistic aggregation controller. For more detailed surveys, including online evolution for single and multi-robot systems, see Bredeche et al. (2018); Francesca and Birattari (2016).

Embodied evolution still faces several challenges in the context of the design of control software for robot swarms. For example, robots need to execute not only their own control software but also the design process. This may not be feasible for robots with limited computational hardware. Furthermore, to conduct the evolutionary process, the swarm must operate for a relatively long time (as compared to the normal mission duration), posing more demand on batteries and increasing the likelihood of sensor or actuator failures. Additionally, without further safety measures implemented *a priori*, the robots risk to damage themselves or the environment, especially in early parts of the design process. More importantly, the evolutionary process can only be achieved if the individuals of the swarm can assess the performance of their chosen genome—ideally this should be computed for the whole swarm, however, without further infrastructure this information is not directly available to the robots as they rely only on local perception.

Three main solutions have been proposed to address the aforementioned issue: open-ended evolution, decomposition and simulation-based assessment. In open-ended evolution, the design process is not driven by an explicit objective function. Instead, open-ended evolution ties the survival of an instance of control software to its ability to “reproduce.” Over time, instances of control software that successfully reproduce will replace instances of control software that cannot. Implicit selection pressure can be exerted by tying the chance to reproduce to certain desired actions or outcomes (Bianco and Nolfi, 2004; Prieto et al., 2010; Bredeche et al., 2012). For example, Bianco and Nolfi consider encounters between robots as opportunity for reproduction. As the task is self-assembly, this implicitly rewards instances of control software that manage to encounter and assemble with other robots. Another typical choice is to model the performance of the individual robots by energy levels: taking an action depletes the energy, but certain outcomes of the actions replenish it. While the robot is active (with available energy), its control software is periodically exchanged with neighboring robots. Once the energy is fully depleted, the instance of control software that was active in the robot is replaced by another one. Over time, instances of control software that are more successful at managing their energy level (by achieving the desired outcomes) will have more opportunities to spread to other robots, thus prevailing in the swarm and displacing less successful instances of control software. Like novelty search, open-ended evolution does not necessarily aim to generate a particular behavior, but rather for the spontaneous emergence of complex behaviors. As an alternative, a designer could manually decompose the objective function for the desired collective behavior into rewards for the actions (or their outcomes) of individual robots (Silva et al., 2015). Although viable, this decomposition is especially difficult in tasks that strictly require cooperation or only provide delayed rewards—e.g., taking an action does not immediately increase the fitness of the swarm, as it requires an appropriate second subsequent action to effectively increase the fitness. This decomposition is similar to the credit assignment problem encountered in robot learning (see Section 4). Recently, Jones et al. (2019) proposed an online evolutionary method in which robots performed simulations to evaluate the quality of genomes. This method allows the robots to estimate the performance of a genome as if it was deployed to the whole swarm—without the need for decomposing the objective function. However, assessing the performance in simulation might overestimate the degree of cooperation and coordination of the robots, as other members of the swarm might execute different instances of control software and not cooperate as expected.

## 4 Robot learning

Reinforcement learning is a method for producing control software in which an agent attempts to learn a policy that encodes the set of optimal actions in a dynamic environment (Kaelbling et al., 1996). Classically, reinforcement learning only considers a single agent interacting with the environment. In this case, the system is then often modelled as a Markov decision process. As robot swarms are composed of several individuals, they are usually modelled as *multi-agent reinforcement learning* problems (see Section 4.1). Robot learning in swarm robotics faces similar challenges as robot evolution. Namely, *reward shaping*, the problem of specifying an appropriate reward function to generate the desired behavior, and the *reality gap*, the drop in performance observed when the control software is designed in simulation and assessed in reality. In multi-agent reinforcement learning methods, all members of the swarm typically act independently. Therefore, they are often affected by the *curse of dimensionality*, where the action space grows with the number of robots and the degrees of freedom of each robot. A variant of reinforcement learning that has found recent application in swarm robotics is *imitation learning* (see Section 4.2). Instead of optimizing the rewards gained from a known reward function, imitation learning aims to imitate a demonstrated behavior.

For reviews of robot learning in the single and multi-robot domain, see Kober et al. (2013); Zhao et al. (2020).

### 4.1 Multi-agent reinforcement learning

*Multi-agent reinforcement learning*: Robots are controlled by an instance of control software in an arbitrary form. A reinforcement learning algorithm is used to optimize the instance of control software according to a mission-specific reward function. The design process results in a single well-performing instance of control software.

Although multi-agent reinforcement learning has been largely studied in the literature, it has seen little application in swarm robotics so far. The first application of reinforcement learning in a swarm robotics scenario is possibly the one of Matarić. Matarić studied reinforcement learning with a swarm of 4 robots that perform a foraging mission (Matarić, 1997). In a follow-up work, Matarić introduced robot communication in the swarm to synchronize rewards between the robots (Matarić, 1998). More recently, Hüttenrauch et al. (2019) used deep reinforcement learning to generate control software for a swarm of virtual agents. Bloom et al. (2022) investigated the use of four deep reinforcement learning techniques in a collective transport experiment.

The application of multi-agent reinforcement learning poses several challenges that still hinder its application in swarm robotics. A first challenge arises from the fact that, in swarm robotics, the desired behavior is usually expressed at the collective level, whereas the learning must happen at the individual level. Thus, when designing control software using reinforcement learning, the mission designer needs to decompose the reward function of the whole swarm into rewards that can be assigned for individual contributions. This problem is also known as *spatial credit assignment*. To this date, no generally applicable methodology exists to address this problem and most works use manual credit assignment (Matarić, 1998; Hüttenrauch et al., 2019; Bloom et al., 2022).

Another important issue is the representation of the state and action spaces in the learning process. Typically, a multi-agent reinforcement learning uses joint action and state spaces, which are concatenated over the individual action and state spaces of each individual agent. These joint spaces, however, suffer heavily from the curse of dimensionality, as they scale poorly both in the size of the individual spaces and in the number of agents. Consequently, addressing large swarm sizes is infeasible in practice. Furthermore, the joint space is not observable by any individual agent, due to the locality of information in a robot swarm. In this sense, the problem of multi-agent reinforcement learning for swarms is more correctly modelled by a partially observable Markov decision process (Kaelbling et al., 1996). In the literature, two techniques have been mostly used to overcome the partial observability: reducing the joint action and state space to those that are pertinent to a single robot (Hüttenrauch et al., 2019; Bloom et al., 2022); or sharing information to synchronize the state beliefs of all members of the swarm (Matarić, 1998). In the first technique, a robot has no model of the behavior of its peers and they are assumed to be part of the environment. The environment that a robot experiences is therefore non-stationary; the state transitions depend not only on the actions of the individual robot but also the (changing, due to learning) behavior of its peers. In the second technique, the swarm retains some level of homogeneity by sharing information between robots. Thus, the learning process does not run independently for each robot but requires some mechanism for synchronization. When using simulations, the centralized-learning/decentralized-execution approach can be used (Hüttenrauch et al., 2019; Bloom et al., 2022). In this approach, observations of all robots are collected centrally and used to update the policy during the learning phase. However, the policy is executed decentralized on each individual robot.

### 4.2 Imitation learning

*Imitation learning*: Given demonstrations of a desired behavior, an algorithm generates an instance of control software that performs the desired behavior. Different techniques, such as behavior cloning, Turing learning, and imitation learning, have been proposed to imitate the demonstrated behavior.

A research field in reinforcement learning that has become of interest for swarm robotics researchers is imitation learning (Osa et al., 2018). In imitation learning, the reward function is assumed to be unknown. Instead, the learning process has access to demonstrations of the desired behavior. The agents attempt to learn a policy that results in a behavior that is similar to the behavior that has been provided in a demonstration. By its own nature, imitation learning methods face a similar challenge as those of novelty search (see Section 2.3). Namely, how to represent a behavior numerically and how to quantitatively measure the similarity of two behaviors.

*Turing learning*: The robots are controlled by an artificial neural network. Demonstrations are used to learn another artificial neural network that discriminates between the demonstrated behavior and the generated ones. The design process iterates between generating an instance of control software and updating the discriminator. New instances of control software are generated in such a way that they can fool the discriminator. Afterwards, the discriminator is updated to once more correctly distinguish between the demonstrated behavior and previously generated ones. The design process results in a single instance of control software, that is behaviorally similar to the demonstrated behavior, and the learned discriminator.

Within the research on imitation learning and swarm robotics, Li et al. (2016) proposed Turing learning for swarm systems. Inspired by the Turing test, the system learns two programs. A first program controls the robots in the swarm, whereas the second program attempts to distinguish between trajectories from the originally demonstrated behavior and trajectories from the behaviors that are being generated through learning.

*Behavior cloning*: Robots are controlled by an instance of control software in an arbitrary form. Demonstrations are used to learn an instance of control software that, under the same conditions, behaves the same as the demonstrated behavior. The reward function computes the similarity of the generated behavior with regard to the demonstrated one. The design process results in a single instance of control software that closely reproduces the demonstrated behavior.

Alharthi et al. (2022) used video recordings of simulated robots to learn a behavior tree corresponding to the demonstrated collective behavior. The authors measure several swarm-level metrics, such as center of mass or length of communication paths in the swarm, and use the Jaccard distance to compute similarity with the original behavior.

*Inverse reinforcement learning*: Robots are controlled by an instance of control software in an arbitrary form. Demonstrations are used to learn the reward function that would be maximized by the demonstrated behavior. The design process results in the learned reward function, which can then be used to learn an instance of control software for the robots.

Šošić et al. (2017) used inverse reinforcement learning to learn the behavior of two predefined particle models. Using SwarmMDP (a variant of decentralized, partially observable Markov decision processes), they reduced the multi-agent reinforcement learning problem to a single-agent problem. Gharbi et al. (2023) used apprenticeship learning (Abbeel and Ng, 2004) to learn collective behaviors from demonstrations of desired spatial organizations for a swarm.

Few studies have focused on the application of imitation learning in swarm robotics. Methods that are being currently developed face two challenges. The first challenge is that existing methods typically require detailed demonstrations to produce their corresponding control software. The more detailed the demonstrations, the easier it is to imitate them. Most work on imitation learning in swarm robotics uses an already available behavior to generate trajectories that must be learned again by the swarm (Li et al., 2016; Šošić et al., 2017; Alharthi et al., 2022). The obvious drawback of this approach is that it is only suitable for cases in which an implementation of the desired collective behavior already exists. Alternatively, other approaches have focused on only demonstrating a few key elements of the collective behavior, instead of a full trajectory (Gharbi et al., 2023). The second challenge is that there is no well-established method to measure the similarity between a demonstrated behavior and a generated one. As in novelty search (see Section 2.3), a collective behavior can be described by several possible forms of representations; with both characteristics at the collective and local level. The definition of characteristics at the collective level requires less domain-specific expertise to decide on, but their mathematical formulation is challenging. Individual characteristics are comparably simpler to compute, yet decomposing the desired collective behavior into its individual parts requires prior knowledge of the mission at hand.

As discussed in this section, few studies in swarm robotics have considered robot learning but have shown it to be a viable alternative to robot evolution for the design of robot swarms. Among these studies, multi-agent reinforcement learning aims to learn policies for a given reward function. Yet, like robot evolution, it faces two major challenges: reward shaping (the corresponding problem to fitness engineering) and the reality gap. Imitation learning, conversely, produces control software by imitating a demonstrated desired behavior without the reward function being known. However, it faces a similar challenge as novelty search; it relies on computing behavioral similarity (as opposed to behavioral novelty in novelty search).

## 5 Perspectives

As highlighted in the previous sections, the research on the design of control software for robot swarms has resulted in many promising automatic design methods. Yet, several important challenges remain. In this section, I discuss the issues that affect broad categories of automatic design methods. Additionally, I give an outlook on techniques and methods that I believe could become useful in addressing the challenges in the automatic design of control software for robot swarms.

*How can we develop design methods that are robust to the reality gap?*

While offline design has shown many promising results, a major challenge remains in the issue of the reality gap. Several approaches have been proposed to reduce the effects of the reality gap. For example, *system identification* can be used to develop more realistic simulators that intend to minimize the differences between simulation and reality (Bongard and Lipson, 2004; Zhao et al., 2020). However, this approach is unlikely to succeed (Jakobi, 1997), especially in swarm robotics. First, a simulation can never be identical to the system that is being simulated (Jakobi, 1997). Although investing more resources to make high-fidelity simulations more accurate can indeed reduce the effects of the reality gap to some extent. Performing these high-fidelity simulations for up to thousands (or possibly more) individual robots would require more computing power than can reasonably be provided at the moment. Second, the robots in a swarm are relatively simple and some fault or inaccuracy in their sensors and actuators is acceptable, or even expected. Consequently, if the simulation accurately models the inaccuracies of each robot, the robots of the swarm would not be longer interchangeable: different robots are modeled to behave differently under the same circumstances. This assumption would negatively impact the desirable properties of a robot swarm. In a more general sense, research has shown that the reality gap does not affect all design methods equally and can lead to rank inversions across design methods. Indeed, a design method can perform better in simulation but worse in reality when compared to another design method (Ligot and Birattari, 2020). Therefore, I contend the focus should be on developing design methods that are inherently robust to the reality gap.

In evolutionary swarm robotics, it is often assumed that no or very little domain knowledge exists. Consequently, control software is commonly generated from scratch. However, in many tasks, there exists a reasonable amount of prior domain knowledge that could be used to ease the design process. Automatic modular design allows to incorporate this domain knowledge in the form of software modules (Birattari et al., 2021). Furthermore, the transferability of individual modules can be tested during the conception of the design method. However, the correct introduction of prior domain knowledge remains dependent on the expertise of the designer. Future research will therefore need to consider automatic modular design methods in which the modules are conceived in a mission-agnostic way; thus reducing the dependency on mission-specific domain knowledge.

When no domain knowledge is available *a priori*, other methods could be used. For example, other possible approach could be to periodically assess the transferability of the generated control software during the design process (Koos et al., 2013). The design method will therefore solve a multi-objective optimization problem, in which both the performance in simulation and in reality are considered. Assessing the control software in reality is expensive in comparison to the assessment performed in simulation. Therefore, such a design method would need to also select which instances of control software are assessed on physical robots. An often-made assumption is that control software that results in similar behaviors in simulation will transfer similarly well into reality. Starting from this assumption, one could possibly restrict the assessment of control software on real robots to instances that are behaviorally novel^2^. Ideally, the assessment should be further limited to promising solutions that have the potential to perform well in reality. However, the overly strict application of this idea might result in design methods that risk overlooking solutions that perform worse in simulation but transfer well into reality.

A further improvement could be to include a secondary simulation context (*pseudo-reality*) to assess the transferability of the control software. Ligot and Birattari have shown that the effects of the reality gap can be reproduced in simulation-only experiments (Ligot and Birattari, 2020). If combined with other transferability techniques, pseudo-reality could offer an inexpensive context to quickly assess the transferability of many instances of control software. Periodically, some instances of control software are assessed on real robots to validate the results of the pseudo-reality context and to refine it, if necessary.

*How can we create online design methods for robot swarms?*

Online design does not face the reality gap problem, which makes it an interesting research direction. However, the online design of robot swarms still faces several challenges. Most notably, the robots in the swarm require a way to assess their own performance solely on the local information available to them.

Another important challenge is how to avoid endangering the robots while they are interacting in an unknown environment, without the need to implement safety features *a priori*. Ideally, one could imagine that a rather general baseline behavior (or a set of baseline behaviors) is designed offline in simulation. Once the swarm is deployed in the mission environment, the swarm would then use the baseline behavior as a starting point to design its control software for the specific mission. In the simplest case, the swarm might perform a tuning of the parameters of the control software to counteract the reality gap. In more advanced scenarios, the robots might choose and combine from a set of baseline control software to find an appropriate behavior for the mission on-the-fly, and then fine-tune the parameter of the resulting control software.

*How can we design control software for complex missions?*

While robot swarms have already been successfully employed in a wide variety of common abstract missions, these missions usually are too simple when compared to real-world applications. More complex missions could include those with multiple, possibly conflicting objectives, or missions with dynamic environments. It is well understood that the shaping of reward and objective functions is critical, as the design process is likely to exploit any unintended local optima of the objective function (Divband Soorati and Hamann, 2015; Silva et al., 2016). Further study is required to develop engineering techniques and patterns to define appropriate objective functions.

Alternatively, incremental evolution (Gomez and Miikkulainen, 1997) and curriculum learning (Bengio et al., 2009) might find application in swarm robotics. In these approaches, a complex task is decomposed into simpler ones. The design process designs control software in increasingly complex tasks, using the previously found instances of control software as starting points for the following designs.

Orthogonal to previously mentioned approaches is (cooperative) co-evolution (Nolfi and Floreano, 1998), in which multiple subgroups of robots are evolved independently of each other to cooperate or compete in the same environment. While it is not an approach directly applicable to homogeneous systems, this approach could be beneficial for the design of heterogeneous robot swarms.

*How can we design control software from other mission specifications than objective functions?*

As mentioned before, the choice of an objective function is not straightforward. With the problems of bootstrapping and deception present, the designer of an objective function requires not only domain knowledge of the task at hand, but also expertise in modeling collective behaviors as mathematical functions.

Novelty search has shown promising results to overcome some of these issues, however, it might lack some of the domain knowledge that can be introduced through the objective function, and that is required to obtain good performing control software. Quality-diversity algorithms combine the search for well-performing solutions commonly found in robot evolution with the exploratory search of novelty search (Mouret and Clune, 2015). Other approaches could include open-ended evolution or open-ended learning to generate varieties of different sophisticated swarm behaviors (Stanley et al., 2017; Packard et al., 2019).

Looking beyond swarm robotics, several different approaches in machine learning and evolutionary robotics have moved beyond mission-specific objective functions. For example, large language models are trained to predict the probability that a certain symbol follows the previous sequence of symbols, in an effort to imitate the texts encountered in the training set (Radford et al., 2019; Brown et al., 2020)^3^. Notably, language models are not trained on other desirable objectives except the imitation, such as grammatical correctness, reading comprehension, or trivia knowledge, yet perform well on benchmarks evaluating such objectives (Brown et al., 2020). In the context of swarm robotics, imitating examples obtained from nature might be also a viable approach to designing collective behaviors. Early works in the field were inspired by swarms in nature and often aimed at engineering artificial swarms that behaved similarly. Using imitation learning, it could be possible to automatically learn collective behaviors from swarms found in nature. Additionally, in single robot systems, another research direction makes use of demonstrations provided by human teachers (Osa et al., 2018; Krishnan et al., 2019). If these notions are applied to swarm robotics, a human teacher could demonstrate a desired collective behavior that is then used to learn the individual behavior of the robots.

## 6 Conclusion

The design problem in swarm robotics arises from the complexity of predicting the numerous interactions of the robots at design time. Automatic design of control software has shown to be a promising approach to tackle the design problem. However, it faces two major challenges: overcoming the reality gap and engineering appropriate objective functions. In this work, I first presented recent advances in the automatic design of control software for robot swarms. After, I discussed shortcomings of proposed approaches and provided perspectives on how to possibly overcome those.

## Footnotes

1 Classically, a robot swarm is a homogeneous system—i.e., all robots have the same capabilities and execute the same software. There have been examples of heterogeneous robot swarms (Dorigo et al., 2013), in which parts of the swarm are specialized in such a way that their role cannot be performed by some of the other robots in the swarm. Yet, in these examples, heterogeneous swarms are also redundant to some degree, as each role has at least several robots being able to perform it.

2 Similar considerations as for novelty search and imitation learning—regarding what metrics can be used to characterize a behavior—apply also to this criterion.

3 In the context of language models, a symbol is not necessarily only a character but could also be a representation of any other syntactic element, such as words.
